# Cardiometabolic index and modified cardiometabolic index are associated with early neurological deterioration in patients with acute ischemic stroke

**DOI:** 10.3389/fneur.2026.1817627

**Published:** 2026-05-04

**Authors:** Li Xu, Fan Liu, Xiaoran Zhang, Zhe Xie, Wenwei Zou, Mengchao Wang, Zhongwen Zhi, Yufeng Liu, Liandong Zhao

**Affiliations:** Department of Neurology, The Affiliated Huai’an Hospital of Xuzhou Medical University, The Second People’s Hospital of Huai’an, Huai’an, China

**Keywords:** acute ischemic stroke, cardiometabolic index, early neurological deterioration, modified cardiometabolic index, stroke

## Abstract

**Background:**

Early neurological deterioration (END) in patients with acute ischemic stroke (AIS) leads to a poor prognosis. Previous studies suggest a high risk of END associated with obesity and metabolic abnormalities. The primary aim of this study was to determine if cardiometabolic index (CMI) and modified CMI (MCMI) are linked to END in patients with AIS.

**Methods:**

This study retrospectively included 563 patients with AIS who had not received reperfusion therapy. Among the participants, 215 (38.2%) were female, with a median age of 69 years (interquartile range: 60–75) and a median baseline National Institutes of Health Stroke Scale (NIHSS) score of 2 (interquartile range: 1–3). According to the TOAST classification, 317 cases (56.3%) were identified as large artery atherosclerosis, 58 cases (10.3%) as cardioembolism, and 188 cases (33.4%) as small-artery occlusion. Patients were classified as experiencing END if their total NIHSS score increased by ≥ 2 points or the motor NIHSS score increased by ≥ 1 point within the first 72 h following admission. Multivariate Logistic regression was used to evaluate whether CMI and MCMI were independently associated with the occurrence of END in AIS patients. Restricted cubic spline (RCS) regression analyzed the nonlinear relationship between CMI, MCMI, and END. Additionally, subgroup analyses were conducted to evaluate the applicability of the findings in different populations.

**Results:**

A total of 123 subjects were identified as having combined END during hospitalization. The CMI and MCMI levels in the END group were significantly elevated compared to the non-END group (*p* < 0.001). Multivariate logistic regression analysis indicated that both high-level CMI and MCMI, when treated as categorical or continuous variables, are independent risk factors for END in AIS patients (all *p* < 0.05). Moreover, subgroup analysis showed that this association was stable in different populations (all *p* for interaction >0.05). The RCS curve showed nonlinear associations between CMI (*p* for nonlinear = 0.048), MCMI (*p* for nonlinear <0.001) and END. The areas under the curves of CMI and MCMI were 0.643 (95% Confidence interval (CI): 0.601–0.682) and 0.665 (95%CI: 0.625–0.704), respectively.

**Conclusion:**

Our study showed that CMI and MCMI at admission were independently associated with END in AIS patients, which could be helpful for early risk stratification of stroke patients.

## 1 Introduction

Epidemiological data indicate that the incidence, disability, and mortality rates of acute ischemic stroke (AIS) are projected to increase by 2030 (1, 2). Besides focusing on the disease itself, managing the complications and secondary risks of AIS is crucial in clinical practice. Despite receiving standardized treatment, some patients experience worsening neurological deficits during the acute phase, known as early neurological deterioration (END). END results in extended hospital stays, higher hospitalization costs, and adversely impacts both short-term and long-term prognoses of AIS patients (3). The exact mechanism of END remains complex and is yet to be fully elucidated by existing studies. Potential contributing factors include hypoperfusion, collateral circulation disorders, progression of *in situ* thrombosis, hemorrhagic transformation, and cerebral edema (4). Current research has identified several risk factors associated with END, such as the National Institutes of Health Stroke Scale (NIHSS) score at admission, age, hypertension, diabetes, and atrial fibrillation (5). Although the pathogenesis and factors influencing END are somewhat understood, reliable prediction methods remain lacking. Given END’s considerable impact on stroke patients, identifying dependable and accessible indicators to assess END risk in AIS patients will aid in tailoring individualized clinical treatment plans.

Metabolic syndrome is a pathological state characterized by metabolic disorders of proteins, fats, carbohydrates, and other substances in the human body. It is a significant risk factor for the onset, progression, and all-cause mortality of AIS (6, 7). Obesity, dyslipidemia, and hyperglycemia are primary components of metabolic syndrome. Numerous studies have extensively demonstrated the strong association between these factors and AIS (8–11). Recent studies have increasingly shown that obesity, glucose and lipid metabolism are associated with a higher risk of END. Body mass index (BMI), a metric for assessing obesity, was significantly and independently associated with the incidence of END (12). Prior research indicates that fasting blood glucose (FBG), diabetes history, glycated hemoglobin, and stress hyperglycemia at admission can predict the occurrence of END. This suggests a strong association between blood glucose variations and END occurrence (13–15). Abnormal lipid metabolism, a critical pathophysiological basis of AIS, also contributes to the onset and progression of END. Elevated triglycerides (TG) and low high-density lipoprotein cholesterol (HDL-C) levels may increase END risk significantly through inflammation and atherosclerosis (AS) (16, 17). These studies underscore the potential connections between obesity, blood glucose levels, lipid metabolism, and END.

The Cardiometabolic Index (CMI) is a novel biomarker combining central obesity with lipid metabolism (18). The formula was derived by multiplying the ratio of waist circumference to height with the ratio of TG to HDL-C. Guo Y et al. (19) recently improved the conventional CMI by incorporating FBG into its calculation. They introduced the Modified Cardiometabolic Index (MCMI), demonstrating its efficacy as a predictor for non-alcoholic fatty liver disease and liver fibrosis. MCMI exhibits superior diagnostic and predictive capabilities compared to CMI. Currently, the relationship between CMI, MCMI, and END remains unclear. The primary objective of this study was to investigate the potential connection between CMI, MCMI and END in AIS patients.

## 2 Materials and methods

### 2.1 Study population

Patients with AIS admitted to the Department of Neurology at the Affiliated Huai’an Hospital of Xuzhou Medical University from June 2024 to February 2025 were included in the analysis.

Inclusion criteria: 1. Patients diagnosed with AIS, confirmed via head computed tomography or magnetic resonance imaging; 2. Age ≥18 years old; 3. Onset time <48 h.

Exclusion criteria: 1. Hemorrhagic stroke or transient ischemic attack; 2. Receive intravenous thrombolysis or endovascular treatment; 3. Severe consciousness disorders, such as drowsiness or coma; 4. Combined with severe mental illness or dementia; 5. Significant heart, liver, or kidney insufficiency, malignant tumors, or autoimmune diseases; 6. Active bleeding or bleeding tendency within 1 year (platelet count <60*10^9^/L or activated partial thromboplastin time >60 s or international normalized ratio >3.0); 7. Non-cooperation with neurological tests; and 8. Incomplete clinical data.

### 2.2 Data collection

Data of patients were collected from clinical databases. This included demographic data, vascular risk factors, clinical characteristics, laboratory data, and drug use after admission. Demographic information included gender, age, height, weight, BMI, waist circumference, and waist-to-height ratio (WHtR). Vascular risk factors included hypertension, diabetes, coronary heart disease, atrial fibrillation, smoking, drinking, and history of stroke. Additional clinical variables encompass systolic blood pressure (SBP), diastolic blood pressure (DBP), NIHSS score at admission, and stroke etiology according to the TOAST classification. Fasting venous blood samples were obtained within 24 h post-admission for analysis, including white blood cell count, hemoglobin, platelet count, FBG, hemoglobin A1c (HbA1c), total cholesterol (TC), TG, HDL-C, low-density lipoprotein cholesterol (LDL-C), and serum creatinine levels.

### 2.3 Calculation formula

WHtR=waist circumference(cm)/height(cm)

CMI=TG(mmol/L)/HDL−C(mmol/L)×WHtR

$$\text{MCMI}=\ln\;[\begin{array}{l} \mathrm{TG}\;(\mathrm{mg}/\mathrm{dL})\times \mathrm{FBG}\;(\mathrm{mg}/\mathrm{dL})\\ /\mathrm{HDL}-C\;(\mathrm{mg}/\mathrm{dL}) \end{array}]\times \text{WHtR}$$

### 2.4 Definition of END

In this study, END was defined as an increase of ≥2 points in the total NIHSS score or ≥1 point in the motor NIHSS score within the first 72 h following admission (20).

### 2.5 Statistical analysis

Continuous variables were represented as means with standard deviations, or medians with interquartile ranges, depending on whether they were normally distributed. Categorical variables were expressed as frequencies (percentages). The statistical significance of differences between END and non-END groups was determined through independent samples *t*-tests, chi-square tests, and Kruskal-Wallis H tests. Participants were categorized into three groups based on tertiles of continuous numerical variables. CMI: Tertile 1 (<0.528, *n* = 188), Tertile 2 (0.528–0.916, *n* = 188), and Tertile 3 (≥0.916, *n* = 187); MCMI: Tertile 1 (<2.809, *n* = 187), Tertile 2 (2.809–3.349, *n* = 189), and Tertile 3 (≥3.349, *n* = 187). The relationships between CMI, MCMI and END were analyzed using multivariate logistic regression. Results are expressed as odds ratios (OR) and 95% confidence intervals (CI). Variables with *p* < 0.05 from the univariate analysis were included in the binary logistic regression. Model 1 is unadjusted; Model 2 adjusts for weight; Model 3 adjusts for covariates including weight, SBP, diabetes, TC, LDL-C, FBG, and HbA1c. As MCMI includes FBG, this variable is not adjusted in the association model 3 between MCMI and END. Receiver Operating Characteristic curve (ROC) was used to determine the cut-off value, sensitivity and specificity of CMI and MCMI for predicting END. Additionally, Restricted Cubic Spline (RCS) regression was employed to explore the shape of the association between CMI, MCMI and END, fitting a restricted cubic spline function with 4 knots (at the 5th, 35th, 65th, and 95th percentiles). Subgroup analysis assessed the robustness of the results across different populations.

All data analysis and image production were performed using SPSS (version 25.0), R software (version 4.3.3), Graphpad prism (version 10.4), and MedCalc (version 22.0). A two-sided *p* value <0.05 was considered statistically significant.

## 3 Results

### 3.1 Baseline characteristics

The study included 563 patients with AIS, with a median age of 69 years (Interquartile range: 60–75). Of these, 348 were male (61.8%) and 215 were female (38.2%). Eventually, 123 patients (21.8%) developed END (Supplementary Figure 1). Table 1 presents the baseline characteristics of patients with and without END. The data indicated that patients with END had higher levels of weight, BMI, waist circumference, WHtR, diabetes prevalence, SBP, FBG, HbA1c, TG, TC, LDL-C, CMI, and MCMI compared to the non-END group, whereas HDL-C levels were lower (all *p* < 0.05). No statistically significant differences were found in the remaining variables between the two groups (Table 1).

**Table 1:** Comparisons of baseline characteristics of patients with and without END.

Characteristic	With END (*n* = 123)	Without END (*n* = 440)	*P*
Male, *n* (%)	74 (60.2)	274 (62.3)	0.670
Age, (years)	69 (58–74)	69 (61–76)	0.381
Height, (cm)	165 (160–172)	167 (160–172)	0.865
Weight, (kg)	72 (64–80)	70 (61.25–76)	0.026
BMI, (kg/m^2^)	25.78 (23.38–28.20)	24.94 (22.89–27.00)	0.007
Waist circumference, (cm)	92 (82–103)	86 (81–93)	<0.001
WHtR	0.55 (0.50–0.63)	0.52 (0.49–0.56)	<0.001
Smoking, *n* (%)	31 (25.2)	88 (20.0)	0.211
Drinking, *n* (%)	23 (18.7)	82 (18.6)	0.987
Hypertension, *n* (%)	92 (74.8)	344 (78.2)	0.427
Diabetes, *n* (%)	62 (50.4)	168 (38.2)	0.015
Coronary heart disease, *n* (%)	23 (18.7)	92 (20.9)	0.591
Atrial fibrillation, *n* (%)	10 (8.1)	44 (10.0)	0.534
History of stroke, *n* (%)	20 (16.3)	49 (11.1)	0.126
TOAST classification, *n* (%)			0.404
Large artery atherosclerosis	74 (60.2)	243 (55.2)	
Cardioembolism	9 (7.3)	49 (11.1)	
Small-artery occlusion	40 (32.5)	148 (33.6)	
Baseline NIHSS, (score)	2 (1–4)	2 (1–3)	0.191
SBP, (mmHg)	153 (139–170)	148 (135–162)	0.015
DBP, (mmHg)	85 (76–95)	85 (75–94)	0.143
White blood cell count, (×109 /L)	7.41 (6.23–8.76)	7.17 (5.78–8.61)	0.197
Hemoglobin, (g/L)	140 (126–148)	138.5 (125–150)	0.819
Platelet count, (×109 /L)	198 (169–236)	209 (171–243)	0.240
Creatinine, (μmol/L)	63.0 (51.1–75.0)	63.0 (54.0–76.7)	0.639
FBG, (mg/dL)	119.65 (94.06–164.34)	99.38 (88.75–132.76)	<0.001
HbA1c, (%)	6.1 (5.5–8.1)	5.8 (5.2–7.1)	0.008
TC, (mg/dL)	184.46 (150.81–232.02)	168.21 (139.99–199.54)	<0.001
TG, (mg/dL)	146.98 (105.37–256.78)	120.42 (87.66–160.27)	<0.001
HDL-C, (mg/dL)	40.22 (34.80–50.66)	42.54 (36.74–51.82)	0.027
LDL-C, (mg/dL)	107.89 (76.18–143.47)	98.22 (71.15–124.13)	0.028
CMI	0.92 (0.55–1.51)	0.64 (0.44–0.97)	<0.001
MCMI	3.39 (2.90–4.15)	2.98 (2.66–3.40)	<0.001
Antithrombotic drug use			0.491
Antiplatelet drugs	115 (93.5)	403 (91.6)	
Anticoagulant drugs	8 (6.5)	37 (8.4)	
Lipid-lowering drugs	122 (99.2)	437 (99.3)	0.878
Antihypertensive drugs	89 (72.4)	326 (74.1)	0.699
Antidiabetic drugs	54 (43.9)	155 (35.2)	0.078

END, early neurological deterioration; BMI, body mass index; WHtR, waist-to-height ratio; SBP, systolic blood pressure; DBP, diastolic blood pressure; NIHSS, national institutes of health stroke scale; FBG, fasting blood glucose; HbA1c, hemoglobin a1c; TC, total cholesterol; TG, triglycerides; HDL-C, high-density lipoprotein cholesterol; LDL-C, low-density lipoprotein cholesterol; CMI, cardiometabolic index; MCMI, modified cardiometabolic index.

### 3.2 Association between CMI and MCMI with END

**Figure 1:**
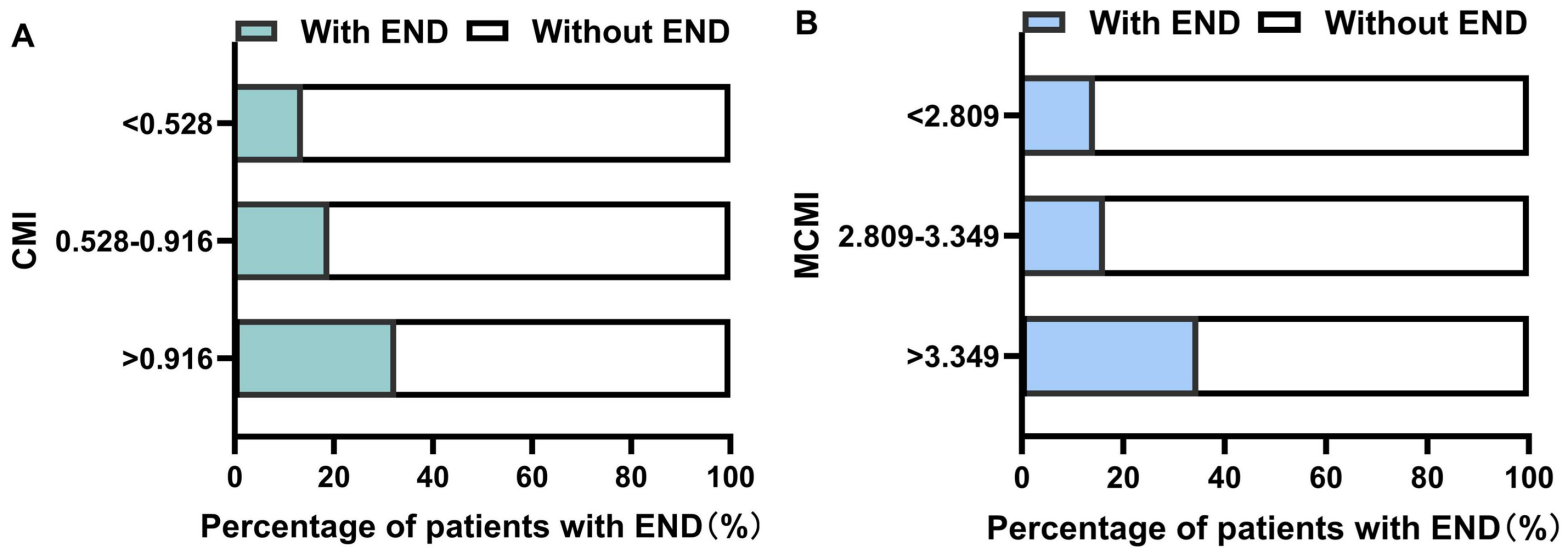
Percentage of END occurrence according to CMI, MCMI tertiles. **(A)** CMI; **(B)** MCMI. END, early neurological deterioration; CMI, cardiometabolic index; MCMI, modified cardiometabolic index.

**Figure 2:**
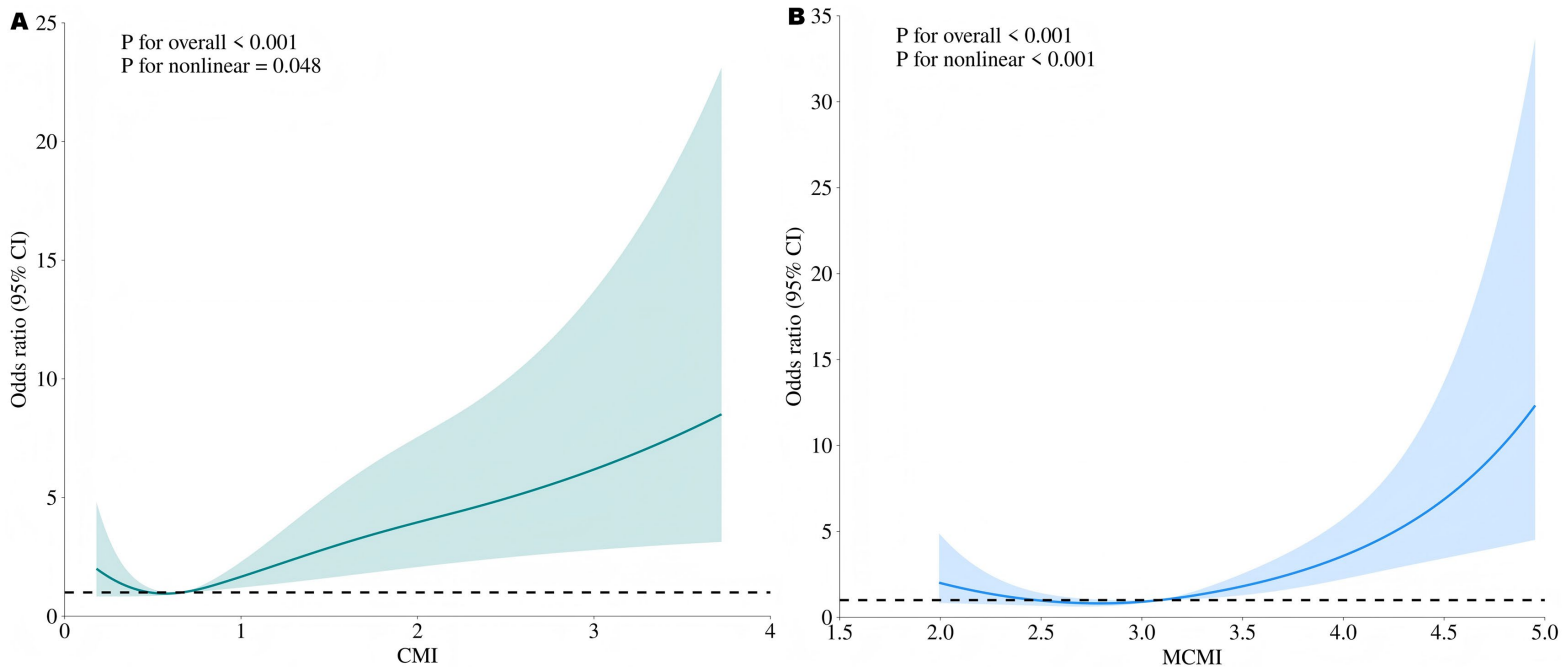
Restricted cubic spline analysis of dose–response relationships between CMI, MCMI and END. **(A)** CMI, adjusted covariates including weight, diabetes, SBP, FBG, HbA1c, TC and LDL-C; **(B)** MCMI, adjusted covariates including weight, diabetes, SBP, HbA1c, TC and LDL-C. END, early neurological deterioration; CMI, cardiometabolic index; MCMI, modified cardiometabolic index.

Subjects were categorized into three CMI tertiles: Tertile 1 (<0.528, *n* = 188), Tertile 2 (0.528–0.916, *n* = 188), and Tertile 3 (≥ 0.916, *n* = 187). The MCMI was grouped similarly: Tertile 1 (<2.809, *n* = 187), Tertile 2 (2.809–3.349, *n* = 189), and Tertile 3 (≥ 3.349, *n* = 187). As the tertiles of CMI and MCMI increased, the prevalence of END significantly increased (all *p* < 0.001) (Figure 1). Table 2 presents the results of the multivariate logistic regression analysis, with Model 1 (unadjusted), Model 2 (partially adjusted), and Model 3 (fully adjusted) outlined. The CMI Tertile 3 group (OR = 2.106, 95% CI: 1.187–3.735, *p* = 0.011) and the MCMI Tertile 3 group (OR = 2.237, 95% CI: 1.199–4.174, *p* = 0.011) exhibited a greater risk of END compared to the Tertile 1 group. When analyzed as continuous numerical variables, and after adjustments in Model 3, elevated CMI (OR = 1.790, 95% CI: 1.321–2.425, *p* < 0.001) and MCMI (OR = 2.482, 95% CI: 1.657–3.717, *p* < 0.001) remained independent risk factors for the occurrence of END (Table 2). The RCS analysis demonstrated a nonlinear correlation between CMI and END (*p* for overall <0.001, *p* for nonlinear = 0.048). Similarly, MCMI showed a nonlinear correlation with END (*p* for overall <0.001, *p* for nonlinear <0.001) (Figure 2).

**Table 2:** Association between CMI, MCMI and END by multivariable logistic-regression.

Variable	END	Model 1		Model 2		Model 3	
	*n* (%)	OR (95%CI)	*P*	OR (95%CI)	*P*	OR (95%CI)	*P*
CMI		1.826 (1.439–2.317)	<0.001	1.777 (1.399–2.258)	<0.001	1.790 (1.321–2.425)	<0.001
CMI tertiles							
Tertile 1	26 (13.8)	Reference		Reference		Reference	
Tertile 2	36 (19.1)	1.476 (0.851–2.560)	0.166	1.431 (0.821–2.494)	0.206	1.336 (0.757–2.358)	0.317
Tertile 3	61 (32.9)	3.016 (1.803–5.047)	<0.001	2.822 (1.649–4.829)	<0.001	2.106 (1.187–3.735)	0.011
MCMI		2.571 (1.894–3.488)	<0.001	2.812 (1.989–3.977)	<0.001	2.482 (1.657–3.717)	<0.001
MCMI tertiles							
Tertile 1	27 (14.4)	Reference		Reference		Reference	
Tertile 2	31 (16.4)	1.163 (0.664–2.037)	0.598	1.161 (0.650–2.074)	0.614	1.044 (0.578–1.886)	0.887
Tertile 3	65 (34.8)	3.157 (1.902–5.241)	<0.001	3.149 (1.783–5.560)	<0.001	2.237 (1.199–4.174)	0.011

Model 1: unadjusted; Model 2: adjusted weight; CMI-Model 3: adjusted for weight, diabetes, SBP, FBG, HbA1c, TC and LDL-C; MCMI-Model 3: adjusted for weight, diabetes, SBP, HbA1c, TC and LDL-C. OR, odds ratio; 95% CI, 95% confidence interval; CMI, cardiometabolic index; MCMI, modified cardiometabolic index; SBP, systolic blood pressure; FBG, fasting blood glucose; HbA1c, hemoglobin a1c; TC, total cholesterol; LDL-C, low-density lipoprotein cholesterol.

### 3.3 Subgroup analysis

**Figure 3:**
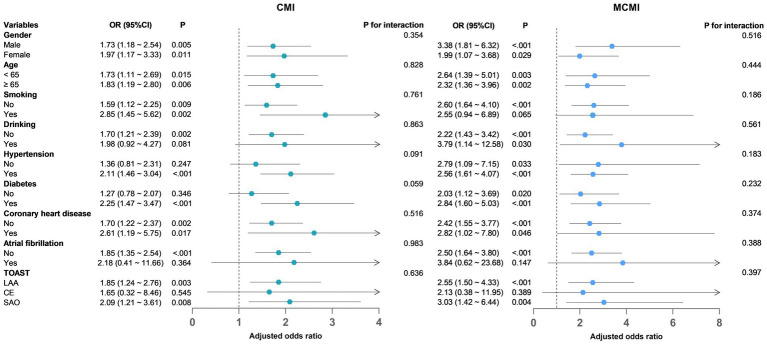
Subgroup analysis of CMI, MCMI and END. CMI, cardiometabolic index; MCMI, modified cardiometabolic index; END, early neurological deterioration.

To evaluate the stability of the association between CMI, MCMI, and END across various populations, subjects were categorized into subgroups based on gender, age, smoking, drinking, hypertension, diabetes, coronary heart disease, atrial fibrillation, and TOAST classification. Adjusted covariates encompassed weight, diabetes, SBP, FBG, HbA1c, TC and LDL-C. Subgroup risk factors and those within CMI and MCMI were not adjusted. The results showed that in all subgroups, no significant interactions were observed between CMI, MCMI and other potential effector moderators (all *p* for interactions >0.05) (Figure 3).

### 3.4 The discrimination ability of CMI and MCMI for END

**Figure 4:**
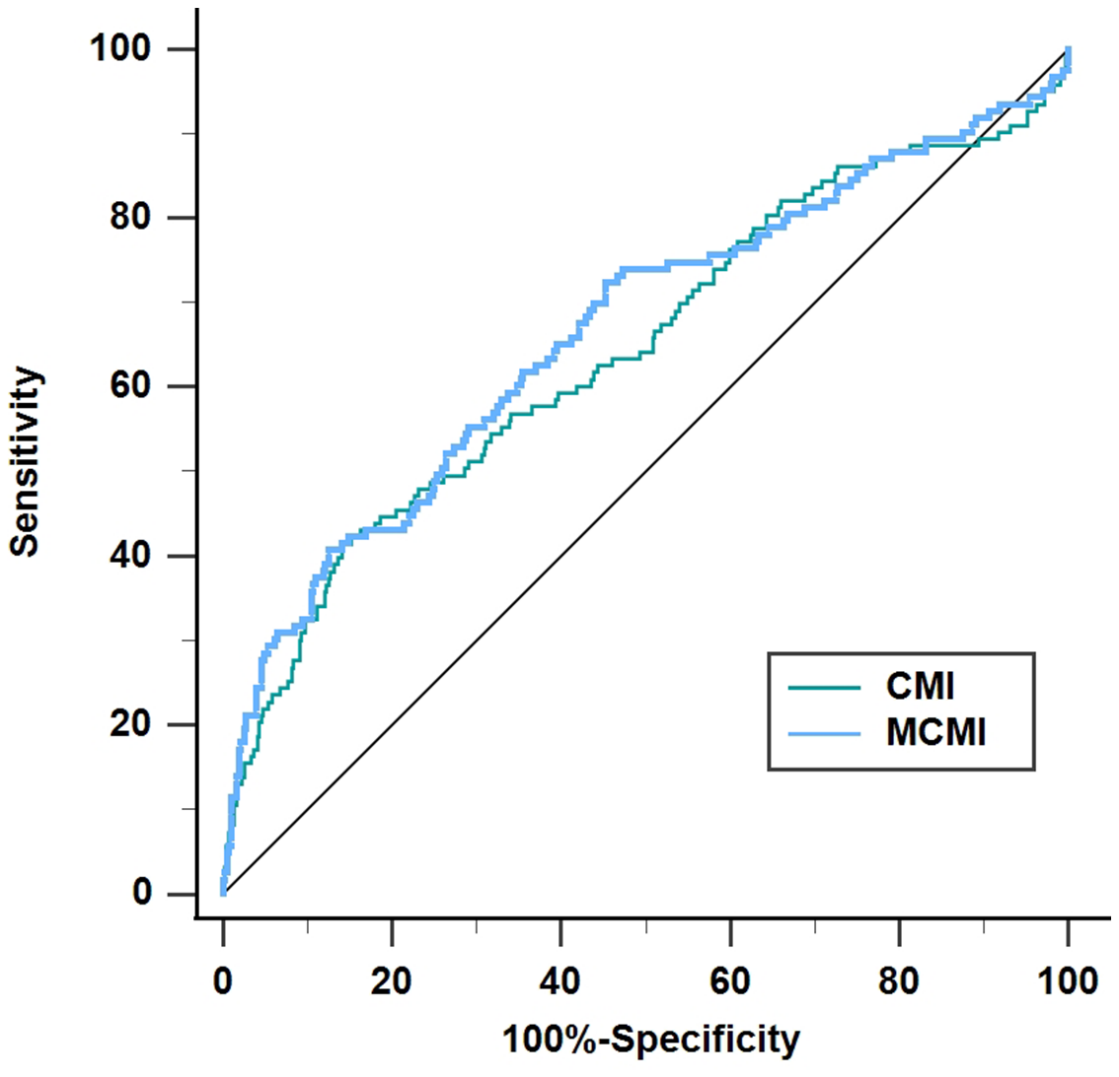
The predictive ability of CMI and MCMI for END. CMI, cardiometabolic index; MCMI, modified cardiometabolic index; END, early neurological deterioration.

ROC curve analysis was used to evaluate the predictive ability of CMI and MCMI for END. The results showed that the Area Under the ROC curve (AUROC) of MCMI was the highest, which was 0.665 (95%CI = 0.625–0.704), and the AUROC of CMI was 0.643 (95%CI = 0.601–0.682). The optimal cut-off value of CMI was 1.165, with a sensitivity of 41.46%, a specificity of 85.68%, and a Youden index of 0.2715. The cut-off value of MCMI was 3.671, the sensitivity was 40.65%, the specificity was 87.50%, and the Youden index was 0.2815 (Figure 4).

## 4 Discussion

Based on prior research (21–23), END have been reported to occur in approximately 5 to 40% of AIS patients. In this study, the incidence of END was 21.8%, aligning closely with earlier findings. Our data shows that CMI and MCMI levels were significantly higher in patients with END. Multivariate analysis indicated that after adjusting for demographic, clinical, and blood covariates, increased levels of CMI and MCMI were independent risk factors for END in AIS patients. Subgroup analysis confirmed the consistency of this association across various population groups. RCS analysis identified significant nonlinear relationship between CMI, MCMI, and END. Additionally, ROC analysis results suggest that CMI and MCMI possess substantial predictive efficacy for END. These findings imply that CMI and MCMI can serve as effective tools for assessing END risk in AIS patients, aiding in the personalized development of clinical treatment strategies.

Obesity is a significant modifiable risk factor for ischemic stroke (IS), with elevated BMI correlating to an increased stroke risk (8). Although BMI is a conventional measure for evaluating obesity, it fails to consider body composition or fat distribution nuances. Consequently, BMI requires reevaluation in the context of etiological analysis within clinical research. WHtR is calculated using height and waist circumference, indicating fat accumulation around the abdomen and central obesity. This measure is more closely associated with hypertension, diabetes, and atherosclerosis (24–26). Zhang P et al. (27) discovered that WHtR had a stronger correlation with stroke prevalence compared to BMI, waist circumference, and waist-to-hip ratio among participants aged 40 years and older. Tian T et al. (12) identified elevated BMI as an independent risk factor for END in AIS patients undergoing intravenous thrombolysis, with notable predictive value. is that obesity amplifies the inflammatory response, subsequently causing endothelial dysfunction and atherosclerosis AS (28, 29). Malnutrition is prevalent among the elderly. Bao Y et al. (30) utilized the Controlling Nutritional Status score, Geriatric Nutritional Risk Index, and Prognostic Nutritional Index to assess the nutritional status of elderly AIS patients aged 65 years or older. Their findings indicated that malnutrition in these patients correlates with the incidence of END. It is noteworthy that this association has not been consistently observed across all age groups. Consequently, the relationship between obesity and END is equally complex, particularly across various populations.

Researchers have extensively investigated the relationship between abnormal lipid metabolism, glucose metabolism, and END. Kwon HM et al. (16) discovered that elevated TG levels (>145 mg/dL) were independently linked to an increased risk of END in patients with acute lacunar stroke. Elevated TG levels can decrease cerebral blood supply by increasing blood viscosity and impeding flow. Moreover, abnormal TG levels may damage vascular endothelial cells, instigate inflammatory responses and AS, and worsen cerebral ischemia and neurological dysfunction, thus contributing to END. HDL-C is considered a protective factor against AS and serves as a potent antioxidant. HDL-C attenuates platelet function by promoting endothelial cell production of nitric oxide and prostacyclin. Additionally, HDL-C enhances blood circulation by inhibiting platelet activating factor and cyclooxygenase A2, thereby preventing thrombosis and platelet activation. Consequently, HDL-C contributes to mitigating oxidative stress during AS development (31). The study found a significant reduction in END in patients within the highest quartile of HDL-C, suggesting that elevated HDL-C levels may protect against END occurrence (17). The atherogenic index of plasma (AIP) was simply calculated from TG and HDL-C. Ki-Woong Nam et al. (32) analyzed 640 patients with large atherosclerotic AIS and found that the AIP helped identify individuals at high risk of END. In patients with AIS undergoing intravenous thrombolysis, fasting blood glucose and the stress hyperglycemia ratio were significantly associated with the occurrence of END (33). Hyperglycemia-induced stress may exacerbate oxidative stress and inflammation, leading to endothelial dysfunction and plaque instability (34). In recent years, alternative indicators of insulin resistance (IR) have been developed based on blood glucose and lipid profiles. For instance, elevated triglycerides-glucose index and the triglycerides-to-high-density lipoprotein cholesterol ratio were regarded as independent predictors of END in AIS patients undergoing intravenous thrombolysis (35). IR can excessively activate platelets, worsen endothelial dysfunction (36, 37), and facilitate atherosclerotic plaque rupture, leading to thrombosis (38). Moreover, IR exacerbates oxidative stress, indirectly worsening cerebral ischemia, reperfusion injury, and inflammatory responses (39, 40).

CMI, initially proposed by Wakabayashi I et al. (18), can be utilized to identify diabetes. It is closely linked to metabolism and serves as an innovative biomarker for cardiovascular disease (CVD) risk factors. By integrating obesity and lipid metabolism, CMI is significantly and independently associated with coronary artery calcification, and it surpasses BMI in predicting coronary heart disease in young and middle-aged adults (41). Data from two prospective cohort studies, the China Health and Retirement Longitudinal Study (CHARLS) and the English Longitudinal Study of Ageing (ELSA), indicate that both CMI and total CMI were linked to an elevated risk of CVD. Compared to those in layer 1, participants in layer 3 exhibited a 53% increase in CVD risk in CHARLS and a 41% increase in ELSA (42). The biological mechanism likely involves the release of pro-inflammatory factors, leading to chronic inflammation and subsequently advancing the progression of AS (43–45). CMI and MCMI integrate data on obesity, lipid and glucose metabolism disorders, which are strongly associated with IR and stroke. Analysis of CHARLS data indicated that, after adjusting for confounding variables, elevated CMI levels were associated with an increased risk of developing hypertension, diabetes, and dyslipidemia (46). A significant association between elevated CMI and increased stroke risk was observed in the Chinese elderly population, and this relationship was nonlinear (47, 48). Our findings indicate that both CMI and MCMI independently correlate with the occurrence of END. The AUROC values of CMI and MCMI are 0.643 and 0.665 respectively, and their sensitivity in predicting END is relatively low. This suggests that the predictive effectiveness of this simplified composite indicator for END is limited. This may be due to the relatively complex mechanism of END, which is not exclusively driven by metabolic abnormalities. In addition, we also analyzed the predictive value of TG, FBG, and BMI for END (Supplementary Figure 2; Supplementary Table 1), it was found that the AUROC of MCMI was the largest among the indicators. Based on these findings, the sensitivity of utilizing CMI and MCMI independently to predict END occurrence appears limited. Nevertheless, this association remains valuable in the risk stratification of stroke patients. In clinical practice, prioritizing high-risk groups through early identification and timely intervention may enhance patient prognosis.

Nonetheless, our study has several limitations. Firstly, due to the differing mechanisms of early neurological deterioration (END) in patients undergoing reperfusion therapy versus those who are not, we excluded patients receiving intravenous thrombolysis and endovascular treatment. Consequently, the findings may only apply to individuals with mild to moderate stroke receiving conservative therapy. Secondly, the single-center nature of the data may limit the generalizability of our findings. Furthermore, variations in fat distribution across ethnic groups necessitate the validation of our findings in prospective studies across diverse regions and ethnicities. Thirdly, blood lipid and glucose levels, key components of CMI and MCMI, may be influenced by lipid-lowering and anti-diabetic medications used prior to stroke. The lack of comprehensive data prevented full control over these variables in the current study. Future research should take into account the impact of these drugs on the research results.

## 5 Conclusion

In conclusion, our study indicates that elevated levels of CMI and MCMI are independent risk factors for END in AIS patients, aiding in the early identification of high-risk groups. Early intervention and tailored treatment strategies could potentially improve outcomes for these patients.

## Data Availability

The raw data supporting the conclusions of this article will be made available by the authors, without undue reservation.

## Supplementary material

SUPPLEMENTARY FIGURE 1 Patient selection flowchart.

SUPPLEMENTARY FIGURE 2 The predictive ability of CMI, MCMI and other indicators for END. CMI, cardiometabolic index; MCMI, modified cardiometabolic index; END, early neurological deterioration.
