# Supplementary figures and images for: Cardiometabolic index and modified cardiometabolic index are associated with early neurological deterioration in patients with acute ischemic stroke
Source: Front Neurol. 2026 May 4;17:1817627. doi: 10.3389/fneur.2026.1817627 (PMC13213422; doi:10.3389/fneur.2026.1817627)

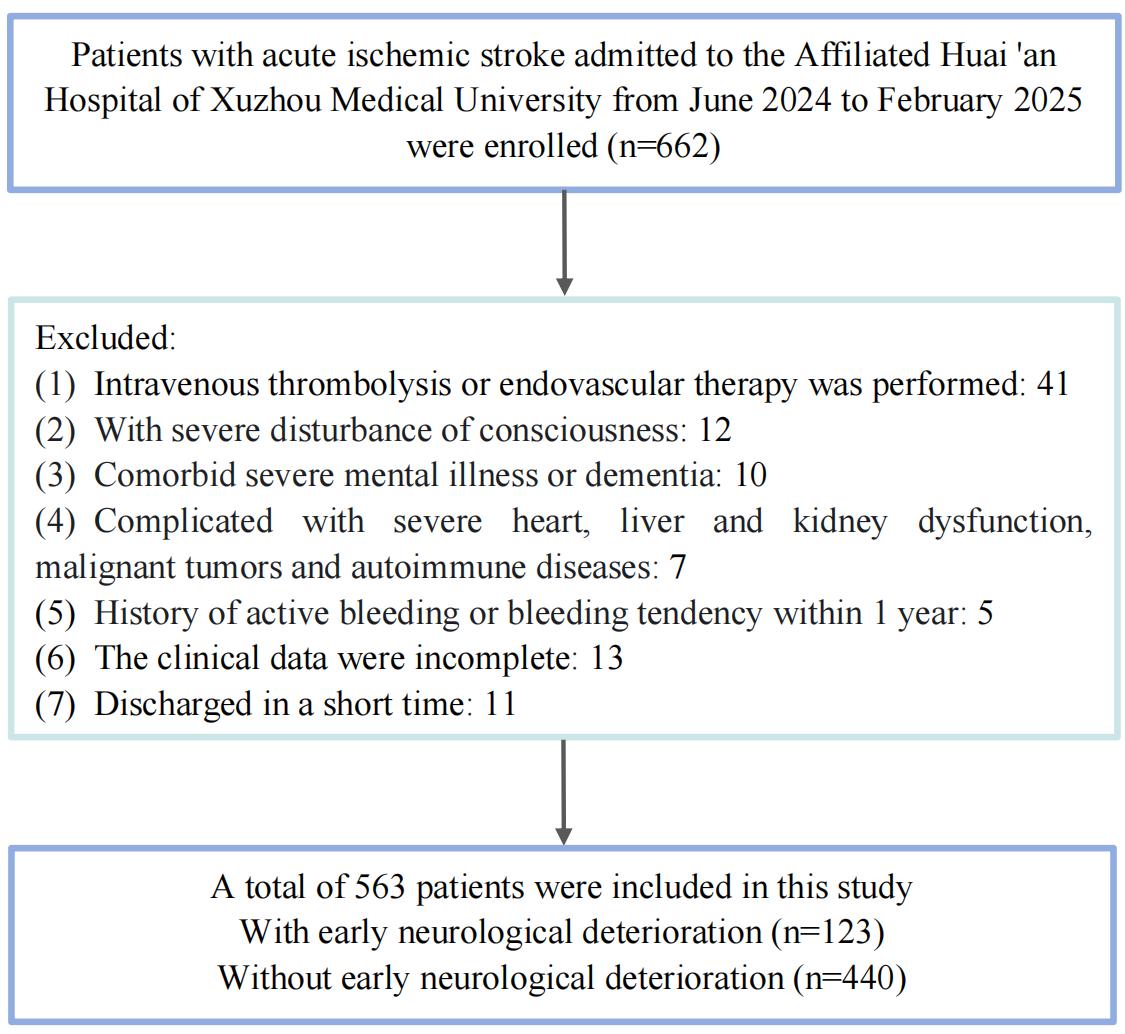

Supplement: SUPPLEMENTARY FIGURE 1 — Patient selection flowchart. [file Image_1.JPEG]

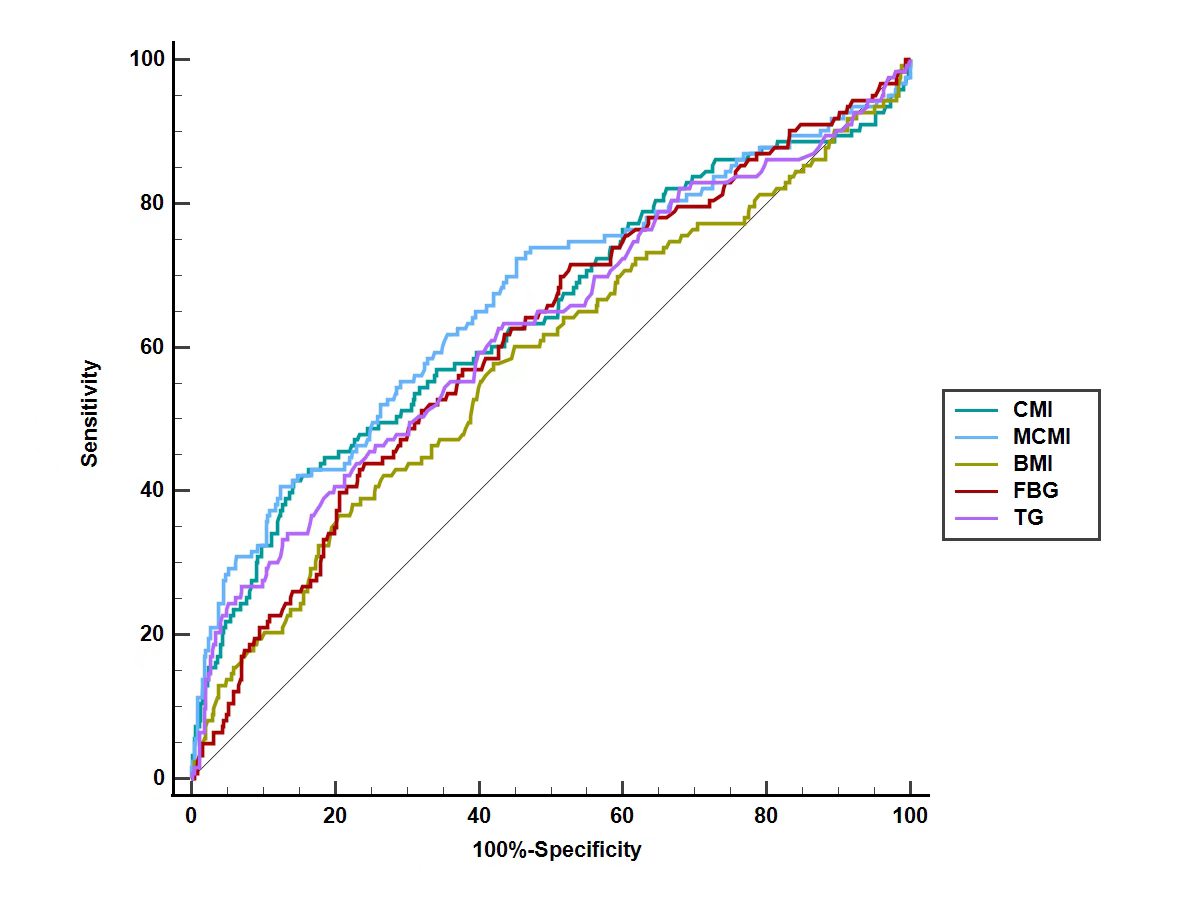

Supplement: SUPPLEMENTARY FIGURE 2 — The predictive ability of CMI, MCMI and other indicators for END. CMI, cardiometabolic index; MCMI, modified cardiometabolic index; END, early neurological deterioration. [file Image_2.JPEG]
