# Supplementary material for: Cardiometabolic index and modified cardiometabolic index are associated with early neurological deterioration in patients with acute ischemic stroke
Source: Front Neurol. 2026 May 4;17:1817627. doi: 10.3389/fneur.2026.1817627 (PMC13213422; doi:10.3389/fneur.2026.1817627)
Supplement: Supplementary file 3 [file Table_1.DOCX]

| **Variables** | **AUROC (95% CI)** | **Cutoff Value** | **Youden Index** | **Sensibility**  **(%)** | **Specificity**  **(%)** | ***P* value** |
| --- | --- | --- | --- | --- | --- | --- |
| CMI | 0.643（0.601-0.682） | 1.165 | 0.2715 | 41.46 | 85.68 | <0.001 |
| MCMI | 0.665（0.625-0.704） | 3.671 | 0.2815 | 40.65 | 87.50 | <0.001 |
| TG | 0.626（0.585 - 0.666） | 166.46 | 0.2095 | 43.90 | 77.05 | <0.001 |
| FBG | 0.613（0.571 - 0.653） | 135.78 | 0.1981 | 43.90 | 75.91 | <0.001 |
| BMI | 0.579（0.537 - 0.620） | 27.43 | 0.1613 | 36.59 | 79.55 | 0.010 |

**Supplementary Table 1** Comparison of Predictive Power of CMI and MCMI Vs Other Indicators in the Prediction of END

Abbreviations: AUROC, Area Under the ROC curve; 95% CI, 95% confidence interval; CMI, Cardiometabolic Index; MCMI, Modified Cardiometabolic Index; END, Early neurological deterioration; TG, Triglycerides; FBG, Fasting blood glucose; BMI, Body mass index.
